# Lipocalin-2 Regulates Osteocyte Ferroptosis and Osteocyte-Osteoblast Crosstalk via Wnt Signaling to Control Bone Formation

**DOI:** 10.21203/rs.3.rs-6430607/v1

**Published:** 2025-04-29

**Authors:** Vivek Khanal, Madeline Carroll, Jayden Carter, Ying Zhong, Shashank Chikkamagaluru, Amy Sato, Ryan Allen, Umesh Wankhade, Neha Dole

**Affiliations:** University of Arkansas for Medical Sciences; University of Arkansas for Medical Sciences; University of Arkansas for Medical Sciences; University of Arkansas for Medical Sciences; University of Arkansas for Medical Sciences; University of Arkansas for Medical Sciences; University of Arkansas for Medical Sciences; University of Arkansas for Medical Sciences; University of Arkansas for Medical Sciences

**Keywords:** Lipocalin 2, Ferroptosis, Mitochondria, Wnt, SLC22A17, Bone

## Abstract

Osteoporosis is a multifactorial disease, and emerging evidence suggests that iron overload contributes to its progression. Here, we identify Lipocalin-2 (LCN2), a cytokine secreted by bone cells with endocrine effects on other tissues, as a local regulator of osteocyte iron metabolism and a mediator of skeletal deterioration. Our findings reveal that LCN2 promotes iron accumulation, mitochondrial dysfunction, and ferroptosis in osteocytes in a process dependent on LCN2 receptor SLC22A17. Genetic ablation of *Lcn2* (*Dmp1*-Cre; *Lcn2*^*fl/fl*^) in osteocytes mitigates their ferroptotic vulnerability by preserving mitochondrial integrity and limiting iron overload. Remarkably, LCN2 deletion enhances osteocyte dendricity and lacunocanalicular network, supporting their function in bone remodeling. Mechanistically, we demonstrate that *Lcn2* ablation in osteocytes decreases DKK1 and SOST expression in bone, leading to increased Wnt/β-catenin signaling and osteoblast-driven bone formation. Using in vitro and in vivo approaches, we establish the LCN2-SLC22A17 axis as a key pathway linking iron homeostasis, osteocyte dysfunction, and skeletal remodeling. These findings provide insight into a previously unrecognized mechanism underlying iron-driven bone loss and suggest that targeting LCN2 could offer therapeutic potential for osteoporosis.

## Introduction

Osteoporosis, characterized by increased susceptibility to fractures and deterioration of bone microarchitecture, remains a critical global health concern^1^. Emerging evidence suggests that excessive iron accumulation significantly contributes to various forms of osteoporosis. Clinical studies have established a heightened prevalence of osteoporosis in conditions associated with chronic iron overload, including thalassemia, sickle cell disease (SCD), and hereditary hemochromatosis (HH)^6–11^. Over half of pediatric patients with thalassemia exhibit diminished bone mineral density (BMD), with prevalence escalating to 87% in adulthood. Similarly, osteoporosis affects up to 72% of adults with SCD, while approximately one-third of HH patients experience substantial bone mass depletion. Moreover, non-genetic factors such as aging, menopause, chronic liver disease, and recurrent blood transfusions—commonly administered for conditions like burn-induced anemia—are also associated with systemic iron overload and heightened osteoporosis risk^12–17^. These findings underscore systemic iron overload as a pivotal determinant of bone fragility.

At the cellular level, preclinical investigations have elucidated that excessive iron levels provoke the generation of reactive oxygen species (ROS) via the Fenton reaction, instigating oxidative stress that inhibits osteoblast proliferation and differentiation while simultaneously augmenting osteoclast activity^17^. These disruptions collectively impair bone remodeling, weaken the microarchitecture, and heighten fracture susceptibility. Addressing the clinical challenges in managing osteoporosis associated with iron overload necessitates a comprehensive understanding of the molecular mechanisms governing iron uptake in bone cells.

Under physiological conditions, iron predominantly circulates in the blood as transferrin-bound iron (holo-transferrin), which is internalized by cells via transferrin receptor 1 (TFR1)-mediated endocytosis^18^. However, under iron overload conditions, blood iron concentrations exceed transferrin’s binding capacity, leading to the emergence of non-transferrin-bound iron (NTBI)—a heterogeneous and poorly characterized mixture that includes ferric citrate and high-mass iron aggregates^19,20^. Ordinarily undetectable in healthy individuals, NTBI becomes measurable when transferrin saturation surpasses 70%, as observed in HH and thalassemia major^21–24^. NTBI is imported into cells through divalent metalion transporters, including ZIP14, ZIP8, DMT1, and SLC22A17, the latter serving as a receptor for Lipocalin-2 (LCN2)^25^. While recent advancements have shed light on transferrin-mediated iron accumulation in bone cells, the mechanisms governing NTBI transport in bone cells remain poorly understood.

Lipocalin-2 is a secreted glycoprotein primarily recognized for its role in innate immunity, where it binds iron-loaded siderophores in the extracellular space to sequester iron from invasive pathogens^26–28^. Independent of infection, though, LCN2 has been shown to bind to iron-siderophore complexes and facilitate NTBI uptake in several tissues, particularly under conditions of iron overload or high iron demand. LCN2-mediated iron uptake has been implicated in exacerbating oxidative stress, mitochondrial dysfunction, and ferroptosis—an iron-dependent form of cell death—in pathologies such as chronic kidney disease, macular degeneration, and ischemic brain injury^29–31^. However, its specific role in bone cells, particularly osteocytes, remains underexplored.

LCN2 is one of the few osteokines known to influence systemic energy balance. Osteoblast-derived LCN2 has been shown to cross the blood-brain barrier and bind to melanocortin receptors in the hypothalamus to signal satiety, thereby regulating body weight and metabolism^32,33^. However, its role in bone homeostasis remains confounding. Global deletion of LCN2 results in osteopenia, with impaired osteoblast function and reduced bone formation, likely due to metabolic disruptions affecting osteoprogenitor activity^33,34^. Similarly, LCN2 expression was also deleted in pre-osteoblasts and osteoblasts targeted by osteocalcin-cre, which adversely affected energy balance but did not impact bone mineral density. In contrast, LCN2 overexpression—achieved using the Col1-Cre model—also leads to bone loss by suppressing osteoblast differentiation and enhancing osteoclast activity^35,36^. These confounding findings suggest that LCN2’s effects on bone are context-dependent.

Since osteocytes are the most abundant bone cells and central regulators of bone turnover, clarifying LCN2’s function in these cells is particularly important. To address this knowledge gap, we investigated whether osteocytes express LCN2 and whether it functions in an autocrine or paracrine manner to support iron uptake in osteocytes and other bone cells. We demonstrate that LCN2 facilitates iron import in osteocytes through its receptor SLC22A17 and that LCN2-driven iron accumulation contributes to mitochondrial dysfunction, oxidative stress, lipid peroxidation, and altered osteocyte-mediated bone remodeling. The ablation of LCN2 in osteocytes negatively impacts bone mass by regulating osteoblast number and function. These findings reveal a previously unappreciated autocrine/paracrine function of LCN2 in bone, which might have implications for skeletal fragility in aging and/or pathologies of iron overload.

## Methods

### Cell Culture and Treatments.

OCY454 osteocyte-like cells^37^ (obtained from Dr. Paola Divieti Pajevic) were cultured in alpha MEM (αMEM, Gibco; Cat# 12571–063) supplemented with 10% heat-inactivated FBS and 1% Anti/Anti. Undifferentiated OCY454 cells were maintained at 33°C, 5% CO₂ and were routinely tested for mycoplasma contamination. For treatment, cells were seeded and allowed to attach overnight. The following day, cells were treated with recombinant mouse Lipocalin 2 (rmLCN2, 100 ng/ml, R&D Systems; Cat# 1857-LC) for 24–48 hours. Prior to treatment, cells were serum-starved for 1 hour, and treatment media was prepared in low serum medium (0.5% FBS). LCN2 expression was knocked down using shRNA (10 nM, VectorLabs Cat# VP-RNAi-7273) transfection via Lipofectamine 3000 (Invitrogen; Cat# L3000001). The expression of *Slc22a17*, was knocked down using siRNA (10 nM, Dharmacon; Cat# M-059399-01-0005). 16–18 hours post-transfection, cells were treated with recombinant rmLCN2 (L) or control (C) for an additional 24 hours. For iron overload conditions, OCY454 cells were cultured in the presence of ferric ammonium chloride (FAC, 250 μM, Sigma; Cat# 158040) for 24 hours, while rmLCN2 treatment was reduced to 6 hours.

### Seahorse extracellular flux assay.

Undifferentiated OCY454 cells were seeded into specialized XFe96/XF Pro microplates (Agilent Seahorse Bioscience; Cat# 101085–004) at a density of 10,000 cells/well and cultured overnight before treatments^38^. Cells were serum-starved for one hour and then treated with rmLCN2 for 24 hours. Treatments were prepared in 0.5% FBS-αMEM media. Following treatment, cells were subjected to oxygen consumption rate (OCR) and extracellular acidification rate (ECAR) measurements using a Seahorse Bioscience Extracellular Flux Analyzer (Agilent). OCR was measured by sequentially treating cells with oligomycin (Millipore; Cat# 495455–10MG, 2 μM), FCCP (Carbonyl cyanide-p-trifluoromethoxyphenylhydrazone, Sigma; Cat# C2920–10MG, 2 μM), rotenone (Fluka; Cat# 45656–250MG, 1 μM), and antimycin A (Sigma; Cat# A8674–25MG, 1 μM). After the assay, cells were lysed in RIPA buffer, and a protein assay was performed. OCR and ECAR values were normalized to protein concentration in each well using Wave Desktop software (v 2.6, Agilent). Data were obtained from three independent biological experiments, each with at least 6 technical replicates. Representative data from one of the experiments is shown.

### Cellular ROS Analysis.

Intracellular ROS levels were measured using the DCFDA/H₂DCFDA (2’,7’ – dichlorofluorescein diacetate) Cellular ROS Assay Kit (Abcam; Cat# ab113851) following the manufacturer’s instructions^38^. Cells were stained with 20 μM DCFDA in 1X buffer for 30 minutes at 37°C, washed in 1X buffer, and analyzed by flow cytometry on the LSR FORTESSA instrument (BD Biosciences, San Jose, CA; Cat# 647586) using FACS Diva software (BD; Cat# 661618). The final analysis, including the generation of plots and histograms, was performed using FlowJo software v.10 (BD; Cat# 446593). Each experiment included three technical replicates, and reproducible data from three independent experiments is reported.

### Mitochondrial Membrane Potential Analysis.

Mitochondrial membrane potential (ΔΨm) was evaluated using the potentiometric dye JC-1 (5,5′,6,6′-tetrachloro-1,1′,3,3′-tetraethyl benzimidazole-carbocyanine iodide; Carlo Erba, Cat# 235425000)^38^. Cells were seeded on collagen-coated ibidi chambered slides (Ibidi, Cat# 80826) at a density of 40,000 cells per dish, allowed to attach overnight, and then treated for 24 hours. The media was replaced with fresh media containing JC1 (10 μg/ml), and cells were incubated in the dark for 30 minutes at 37°C. After incubation, media containing JC1 was removed, and cells were washed and incubated in reduced serum media (αMEM supplemented with 0.5% FBS). Confocal z-stacks were acquired in a chamber equipped for live-cell imaging (5% CO₂, 37°C) using a Zeiss Airyscan confocal microscope with a 63x/1.4 oil objective. Image analysis was performed using ImageJ (NIH; RRID: SCR_003070) with sum projections of red and green channels. The red-to-green fluorescence intensity ratio (R/G) was calculated by dividing red intensity by green intensity. These values were normalized to the control condition, and the fold change in mean R/G fluorescence was reported. For each treatment, 3–4 images were collected per experiment, and representative data from one experiment is shown. The results were reproducible across three independent experiments conducted by two individuals.

### Lipid Peroxidation Analysis.

Intracellular lipid peroxidation levels were measured using the C11-BODIPY 581/591 kit (Invitrogen; Cat# D3861) following the manufacturer’s instructions^39^. Cells were seeded on 24-well plates (Corning; Cat# 3526) at 40,000 cells per well, allowed to attach overnight, and treated for 24 hours with 100 ng/ml rmLCN2. After treatment, cells were washed with PBS, trypsinized, and stained in α-MEM containing 5 ng/ml C11-BODIPY for 30 minutes at 37°C. Cells were analyzed by flow cytometry using LSR FORTESSA, with the reduced and oxidized forms of C11-BODIPY measured at 581–591 nm and 488–510 nm, respectively. The ratio of oxidized to reduced C11-BODIPY stained cells was evaluated using FlowJo software v.10. At least three technical replicates were used, and reproducible data from three independent experiments is reported.

### FerroOrange Staining.

The labile iron pool was assessed using FerroOrange staining (Dojindo; Cat# F374)^40^. Cells were seeded at 10,000 cells per well in 8-well Ibidi chambered slides (Ibidi; Cat# 80826) and allowed to adhere overnight. The following day, cells were treated with rmLCN2 (100ng/ml) for 24 hours, washed with PBS, and stained in α-MEM containing 1 μM FerroOrange. The cells were incubated in the dark for 30–45 minutes at 37°C, washed three times with Hank Buffered Saline Solution (HBSS, Gibco; Cat# 14025092), and counterstained with 1 μg/ml DAPI (Thermo Fisher; Cat# D1306). Immediately after staining, cells were covered with base media and transferred to a live cell chamber (Tokai Hit; Cat# INUBTF-WSKM-F1, 5% CO₂, 37°C). Confocal images were acquired using a Zeiss LSM880 microscope (Zeiss; Cat# 1024317) with 20x and 40x/1.4 oil objectives (Zeiss; Cat# 420792-9900-000). Fluorescence intensities from 3–4 images per treatment were analyzed using ImageJ (NIH; RRID: SCR_003070) and normalized to the control condition. Representative data from one experiment is shown, with results reproducible across three independent biological experiments conducted by two researchers.

### Cell Death Assessment.

Cell death was assessed using the Annexin V-FITC and Propidium Iodide (PI) staining kit for Flow Cytometry (Invitrogen; Cat# V13242) following the manufacturer’s protocol^38^. Cells were seeded in 24-well plates (Corning; Cat# 3526) at 40,000 cells per well, allowed to adhere overnight, and treated for 24 hours with rmLCN2. After treatment, cells were trypsinized, centrifuged at 3000 RPM for 3 minutes, and reconstituted in 1X Annexin binding buffer (Invitrogen; Cat# V13242). Annexin V-FITC and PI were added, and cells were incubated for 15 minutes at room temperature before analysis by flow cytometry (LSR FORTESSA, BD Biosciences; Cat# 647586). The percentage of apoptotic and necrotic cells was quantified using FlowJo software v.10 (BD; Cat# 446593). Each experiment included 3–4 technical replicates, and data from three independent experiments is reported.

### Western blots.

Whole-cell lysates of OCY454 cells were collected in RIPA lysis buffer containing 50 mM Tris (pH 7.4), 1% NP-40, 0.25% sodium deoxycholate, 150 mM NaCl, and 1 mM EDTA, supplemented with phosphatase inhibitor (Pierce; Cat# A32957), protease inhibitor (cOmplete Mini, Roche; Cat#11836153001), and 1 mM PMSF (Sigma; Cat# P7626)^38^. Lysates were sonicated on ice using a probe sonicator (five 15-second pulses, 45 seconds between pulses) and cleared by centrifugation at 10,000×g for 10 minutes at 4°C. Protein concentration was determined using the Pierce BCA Protein Assay Kit (Thermo Scientific; Cat# 23225). 25 μg of total lysate per sample was loaded onto 10% SDS-polyacrylamide gels, separated by electrophoresis, transferred to a nitrocellulose membrane (LI-COR; Cat# 926–31092), and blocked in Intercept^®^ (PBS) Blocking Buffer (LI-COR; Cat# 927–70001). Membranes were probed with the following primary antibodies prepared in Intercept blocking buffer with0.1% Tween-20: Anti-β-actin (1:2500, Abcam; Cat# ab8226), Anti-β-tubulin (1:1000, Novus Biologicals; Cat# NB100–1612), Anti-Lipocalin 2 (1:1000, Abcam; Cat# ab216462), Anti-DKK1 (1:2000, ProteinTech; Cat# 21112–1-AP), and Anti-active-β-catenin (1:1000, Abcam; Cat# ab305261), Blots were incubated with secondary anti-mouse and anti-rabbit antibodies conjugated to IRDye 680 or 800 fluorophores (1:15,000, LI-COR Biosciences; Cat# 926–68070 for anti-mouse, Cat# 926–32211 for anti-rabbit). Signal detection was performed using the Odyssey infrared imaging system (LI-COR Biosciences; Cat# 9140–00), and band intensities were quantified using Image Studio Lite v5.2 (LI-COR Biosciences). Blots were stripped and re-probed for different antibodies, and the efficiency of the stripping protocol was confirmed by the absence of residual secondary antibody binding. Protein expression was normalized to β-actin or β-tubulin, and fold changes were calculated relative to unstimulated controls. Data is presented as mean ± SD, with blots representative of three independent experiments (N = 3 biological replicates/group/experiment). Ultimately our sample size from compiling 2 independent experiments was 3 biological replicates/group.

### Mouse Husbandry.

Osteocyte- and late-osteoblast-targeted LCN2-deficient mice and littermate controls were generated by breeding *Lcn2* floxed (*Lcn2*^*fl/fl*^; JAX Strain #031034) mice with 9.6kb *Dmp1-Cre* mice (JAX Strain #023047). The resulting offspring included *Dmp1-Cre+/−; Lcn2*^*fl/fl*^ mice (denoted as *Dmp1-Cre; Lcn2*^*fl/fl*^) and *Dmp1-Cre−/−; Lcn2*^*fl/fl*^ littermate controls (denoted as Wild-type, WT Control), which were used for subsequent experiments. Genotyping was performed using 50 ng of genomic DNA extracted from tail biopsies. The presence of the *Dmp1-Cre* transgene was confirmed using the following primers: Forward: 5’-TTG CCT TTC TCT CCA CAG GT-3’, Reverse: 5’-CAT GTC CAT CAG GTT CTT GC-3’. The presence of the *Lcn2* floxed allele was confirmed with the following primers: Forward: 5’-CCT CAA GGA CGA CAA CAT CA-3’ and Reverse: 5’-GAG GAA GCT TGG ACA GGA ATC-3’. Thirteen-week-old WT and *Dmp1-Cre; Lcn2*^*fl/fl*^ male mice were used for the qPCR, bulk RNA-seq immunohistochemistry and staining, bone histomorphometry, and micro-computed tomography. Seventeen-week-old WT and *Dmp1-Cre; Lcn2*^*fl/fl*^ male mice were used for mechanical testing. No randomization was needed as our study groups were based on their genotypes.

### Quantitative real time PCR (qPCR).

RNA was extracted from mouse humeri using the miRNeasy Mini Kit (Qiagen, Valencia, CA) following the manufacturer’s protocol^38,41^. The marrow and periosteum were carefully removed before flash-freezing the bones in liquid nitrogen and homogenizing them in QIAzol. The RNA from cell lysates was extracted using the Direct-Zol RNA Miniprep Kit (Zymo Research). cDNA synthesis was performed using iScript cDNA Synthesis Kit (Bio-Rad) followed by qPCR with iQ SYBR Green Supermix (Bio-Rad) or TaqMan probes (Applied Biosystems, Thermo Fisher Scientific), following the manufacturer’s instructions. β-Actin or 18S ribosomal RNA were used as internal controls for gene expression normalization. The relative mRNA levels were quantified using the 2^−ΔΔCT^ method, and data are presented as fold change in mRNA expression. The primer and probe sequences are listed in Supplemental Table 1.

### Bulk RNA-seq analysis.

Total RNA was isolated from humeri using the miRNeasy minikit (Qiagen, Valencia, CA), and RNA purity was assessed spectrophotometrically (A260/A280 ratio > 1.9). RNA quality was measured via Bioanalyzer (Agilent), where RNA samples with a RIN score greater than 7 were used for library preparation and sequencing. Total RNA was extracted from two pooled humeri per mouse, and equal amounts were used to generate biologically distinct replicates for each group (n = 4 mice/group).

Stranded mRNA-seq libraries were prepared using the NEB-Next Ultra reagents (New England Biolabs). First and second strand cDNA synthesis, end-filling using Klenow fragment, and dA-tailing were performed per manufacturer instructions. Ligations with Illumina paired-end adapters for multiplexed sequencing were carried out in a 50 μL reaction volume for 30 min at room temperature using 1 μL of T4 DNA ligase and 0.3 μM of annealed adapters. Ligated products were size-selected using a high-resolution 2% agarose gel, and fragments around 200 bp (± 50 bp) were excised and purified with the Qiagen Gel Extraction Kit. Size-selected cDNA libraries were amplified using indexed primers for 12–14 cycles in a 50 μL reaction containing 29 μL of template, 1 μL of forward and reverse primers (25 μM), and 1 U of Phusion high-fidelity DNA polymerase (New England Biolabs). PCR products were purified using the Qiaquick PCR purification kit (Qiagen) and eluted in 30 μL of nuclease-free water. A small aliquot (~ 1 μL) was analyzed with a DNA1K chip (Experion, Bio-Rad) to confirm the absence of primer-dimers or spurious products. RNA-seq libraries were quantified using the Qubit dsDNA HS Assay kit.

Libraries were sequenced on the Illumina NextSeq 2000 platform using single-end 100 bp reads. Raw FASTQ files were obtained directly from the Illumina BaseSpace environment following. Files corresponding to each biological sample were downloaded and organized for downstream analysis. Initial quality control (QC) was performed using the DRAGEN FASTQC app within BaseSpace. QC metrics such as per base sequence quality, per sequence GC content, adapter content, and sequence duplication levels were assessed, and only samples meeting QC thresholds were retained for further analysis. To aggregate and summarize QC results across all samples, output from DRAGEN FASTQC was compiled using the MultiQC tool. MultiQC generated a comprehensive report consolidating individual FASTQC metrics, facilitating comparison across the dataset and aiding in the identification of systemic issues or outlier samples. High-quality reads were processed using the DRAGEN RNA Pipeline in BaseSpace. This pipeline performs alignment and quantification using the DRAGEN Bio-IT Platform, offering a hardware-accelerated and highly optimized RNA-seq workflow. Reads were aligned to the appropriate reference genome (GRCm38_v100) using the DRAGEN aligner. Gene- and transcript-level quantification was performed using a GTF annotation file provided with the reference genome. Expression levels were reported as raw read counts, TPM (transcripts per million), and FPKM (fragments per kilobase of transcript per million mapped reads), as applicable.

The DRAGEN RNA pipeline output included aligned BAM files, which were used for downstream differential expression analysis and data visualization. BAM files were analyzed in SeqMonk v1.47.2 for transcript quantification. Genes with fewer than 25 counts were removed, and differentially expressed genes (DEGs) were identified using the DESeq2 algorithm.

Genes with less than 25 counts were removed and differentially expressed genes (DEGs) were identified using the DESeq2 algorithm with bone RNA from WT mice as the control. Genes were considered differentially expressed if they met the following criteria: false discovery rate (FDR) ≤ 0.05 and absolute log2 fold change ≥ 1.5. Ferroptosis-related genes were obtained from the FerrDb database^42^. Additionally, differentially expressed genes were mapped to mitochondrial pathways using the MitoCarta database, identifying 222 significantly upregulated genes involved in mitochondrial function, including pathways related to mitochondrial central dogma, protein import and sorting, protein homeostasis, oxidative phosphorylation (OXPHOS), metabolism, small molecule transport, signaling, and mitochondrial dynamics and surveillance.

### Immunohistochemistry and Staining.

Paraffin-embedded bone sections (5 μm thick) were deparaffinized, rehydrated, subjected to antigen retrieval, blocked, and permeabilized with 0.5% Triton X-100 (Innovex)^38^. Sections were then incubated overnight at 4°C with primary antibodies, including anti-SLC7A11 (1:250, Abcam, ab307601), anti-GPX4 (1:50, Abcam, ab125066), anti-LCN2 (1:250, Abcam, ab216462), anti-MDA (1:200, Abcam, ab243066), anti-SOST (1:100, R&D Systems, BAF1589), anti-DKK1 (1:100, ProteinTech, 21112–1-AP), and anti-active-β-catenin (1:500, Abcam, ab305261). The next day, sections were incubated for 1 hour at room temperature with fluorescent secondary antibodies, including Donkey Anti-Goat IgG (1:1000, Jackson ImmunoResearch, 705-606-147, Alexa Fluor^®^ 647) and Goat Anti-Rabbit IgG H&L (1:1000, Abcam, ab150080, Alexa Fluor^®^ 594). Corresponding nonimmune IgGs (Abcam, ab172730 for rabbit and R&D systems, AB-108-C for goat) were used as negative controls. H&E staining was performed to assess viable osteocytes.

Bone sections were stained for Perl’s Prussian Blue (Abcam, ab150674) to detect Fe3+ deposits, following the manufacturer’s protocol^43^. Sections were incubated with 1:2 HCl- Potassium-Ferrocyanide (Sigma, P-3289) for 30 min, rinsed in distilled water, counterstained with nuclear fast red (Sigma, 60700) for 1 min, and examined by light microscopy. Staining interpretation: nuclei appear red, cytoplasm pink, and iron deposits bright blue.

Osteocyte lacunocanalicular networks were visualized using Ploton silver staining^38,44^, with images acquired on a ZEISS Axio Imager M2 or Olympus BX53 and quantified using ImageJ. 10 μm thick frozen sections were prepared by embedding femurs in O.C.T. Compound (Fisher, 23-730-571) at − 25°C and labeled with phalloidin (Alexa Fluor 594, 5 μg/ml; Invitrogen, R37110) and DAPI (10 μg/ml; Invitrogen, P36935) to stain F-actin and osteocyte networks^45^. Confocal images were acquired using a Zeiss LSM880 microscope with a 100× oil objective. The Simple Neurite Tracer plugin for ImageJ was used to analyze osteocyte dendrite number and length^46^. Z-stack images were converted to greyscale, and dendrites were semi-automatically traced to obtain average length and number per osteocyte. Four mice per group were analyzed, with three images per mouse, assessing 2–3 osteocytes per image within the full field of view.

### Bone histomorphometric analysis.

Dynamic histomorphometry was performed to assess mineralizing surface to bone surface (MS/BS), mineral apposition rate (MAR), and bone formation rate normalized to bone surface (BFR/BS)^47^. Mice received intraperitoneal injections of calcein green (G, 30 mg/kg) and alizarin red (A, 50 mg/kg) in a G-A sequence at 10 and 3 days before euthanasia. Left tibias were fixed in 10% buffered formalin and embedded undecalcified in methyl methacrylate. Thick cross-sections at the mid-diaphysis were prepared using a diamond-embedded wire saw (Histosaw, Delaware Diamond Knives, Wilmington, DE, USA) and ground to a final thickness of 30–40 μm for periosteal and endosteal bone formation measurements. For static histomorphometric analysis, longitudinal sections of distal femurs were stained for TRAPase and counterstained with toluidine blue. TRAPase + multinucleated osteoclasts (≥ 3 nuclei) attached to the cancellous bone region (350–1750 μm from the distal growth plate) were quantified. Histomorphometric analysis was conducted using the OsteoMeasure High-Resolution Digital Video System (OsteoMetrics, Decatur, GA) interfaced with an Olympus BX51 fluorescence microscope (Olympus America Inc., Center Valley, PA). All analyses followed ASBMR Histomorphometry Nomenclature Committee guidelines and were performed in a blinded manner.

### Micro-computed tomography (μCT).

Femurs from 13-week-old male WT and *Dmp1-Cre; Lcn2*^*fl/fl*^ mice were dissected, cleaned of soft tissue, and fixed in 10% neutral buffered formalin for 48 hours before being stored in 70% ethanol for scanning^47^. μCT analysis was performed using the VivaCT-80 (Scanco Medical, Switzerland) with the following settings: E = 70 kVp, I = 114 μA, integration time = 200 ms, and an isotropic voxel size of 10.4 μm. For trabecular analysis, a Gaussian filter (sigma = 0.8, support = 1) was applied, and a lower threshold of 450 mg/cm^3^ was used. Trabecular bone was analyzed in the distal femur beginning 10 slices away from the growth plate to exclude the primary spongiosa, extending for a total of 150 slices. Cortical bone was analyzed using a lower threshold of 580 mg/cm^3^. Mid-diaphyseal cortical bone parameters were assessed over a 40-slice region centered at the femoral midpoint. All nomenclature, symbols, and units adhered to established μCT guidelines, and n = 8–10 mice per group were analyzed.

### Mechanical testing.

Structural and material properties of femurs from 17-week-old WT and *Dmp1-Cre; Lcn2*^*fl/fl*^ male mice were assessed using three-point bending^38,47,48^. Femurs were stored at − 20°C in HBSS-soaked gauze and thawed to room temperature before testing. Testing was conducted on an Electroforce TA 5500 system (TA Instruments, New Castle, DE)^49^ with a loading rate of 3 mm/min and an lower support span of 8 mm that recorded the application force (N) and displacement (mm) in real-time. Femoral length was measured with a digital caliper prior to force application. The polar moment of inertia and minor diameter (anterior to posterior diameter) at the femoral midshaft were determined by μCT (VivaCT-80, Scanco Medical) with a 10um voxel resolution. Mechanical properties were normalized for bone size and used to calculate material properties using standard equations as previously published^50,51^.

### Study approval.

Mice were housed in groups in a pathogen-free facility at 22°C with a 12-hour light/dark cycle and fed their respective diets (irradiated) and water ad libitum. All studies were conducted with the approval of the Institutional Animal Care and Use Committee of the University of Arkansas for Medical Sciences.

### Statistical analysis.

The sample size was determined based on a power calculation that provides an 80% chance of detecting a significant difference (p < 0.05). Technical replicates and biological replicates (n) used for all experiments are described in the figure legends. Data are reported as mean ± S.D. Prism 10.0 (GraphPad Software, Inc.) was used for statistical analysis. Student two-tailed t-tests were used when determining the statistical differences between the two groups; while comparing multiple groups, we used one-way ANOVA followed by the Newman-Keuls test for multiple comparisons. To analyze the effect of two variables and the interaction of those variables, we used two-way ANOVA followed by the Newman-Keuls test for multiple comparisons. Experiments using primary cell cultures were performed with at least three replicates and independently repeated two times. Experiments using OCY454 cells were repeated with 3–4 technical replicates and at least three times by two investigators. Investigators were blinded to the group allocation regarding the genotype of mice during the experiment, and the genotype information was revealed during data interpretation.

## Results

### LCN2 promotes osteocyte ferroptosis.

**Figure 1:**
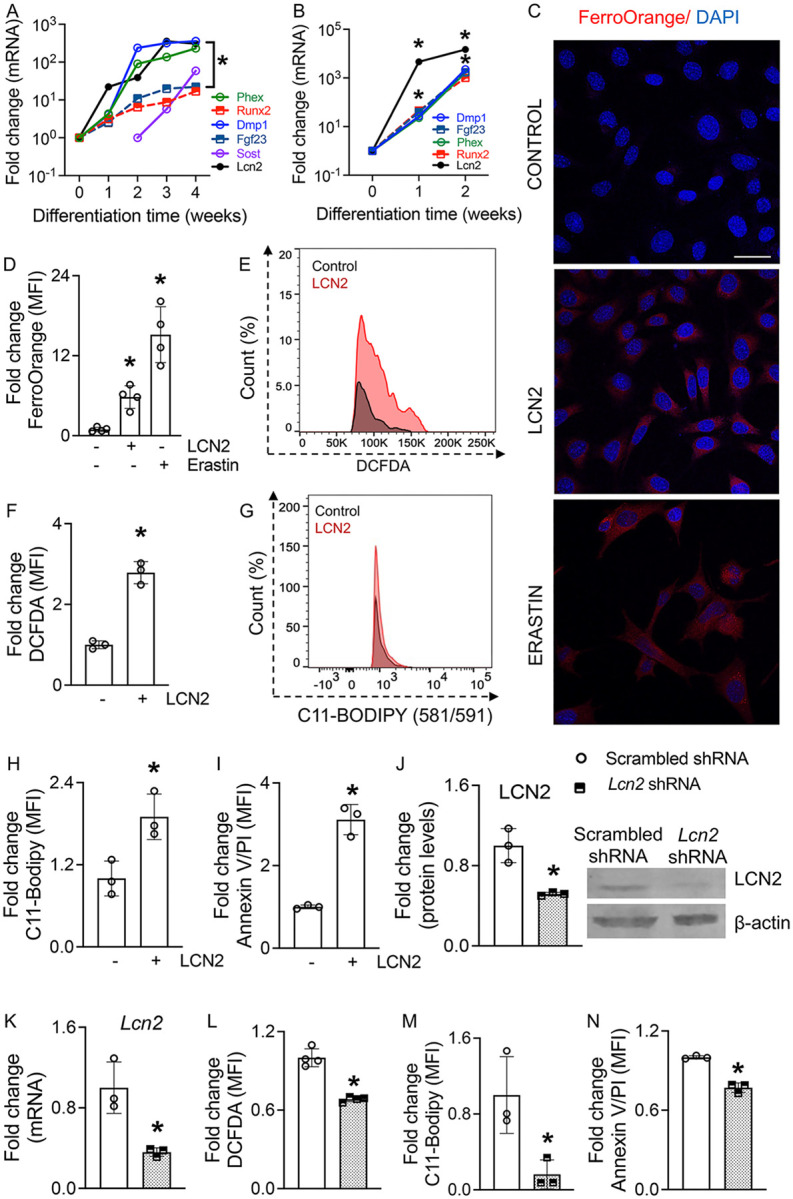
LCN2 promotes ferroptosis by increasing iron accumulation, oxidative stress, lipid peroxidation, and cell death in osteocytes. (A, B) qPCR analysis of *Lcn2*, osteoblast, and osteocyte markers, namely, *Phex, Runx2, Dmp1, Fgf23, Sost* in (A) BMSCs and (B) OCY454 osteocyte-like cells at different time points of osteogenic differentiation. *p<0.05 vs. week 0, n=4 biological replicates/time point. (C, D) FerroOrange staining (C, scale bar = 40 μm) and quantification (D) of Fe^2+^ levels in OCY454 cells treated with recombinant murine LCN2 (rmLCN2, 100 ng/ml) or erastin for 24 hours. n=4 biological replicates/group. (E, F) Flow cytometric quantification of DCFDA fluorescence showing increased oxidative stress (ROS) in OCY454 cells treated with rmLCN2 for 24 hours. n=3 biological replicates/group. (G, H) Flow cytometric quantification of C11-BODIPY fluorescence indicating increased lipid peroxidation in OCY454 cells treated with rmLCN2 for 24 hours. n=3 biological replicates/group. (I) Flow cytometric analysis of Annexin V/PI staining showing increased ferroptotic cell death in OCY454 cells treated with LCN2 for 48 hours. p<0.05 vs. untreated cells, n=3 biological replicates/group. (J, K) Western blot (J) and qPCR (K) analysis showing reduced LCN2 protein and mRNA expression in OCY454 cells transfected with *Lcn2* shRNA compared to scrambled control. n=4 biological replicates/group. (L-N) Flow cytometric quantification of DCFDA fluorescence (L), C11-BODIPY fluorescence (M), and Annexin V/PI staining (N) showing reduced oxidative stress, lipid peroxidation, and ferroptotic cell death in LCN2 shRNA-transfected OCY454 cells. n=4 biological replicates/group. Data are presented as mean ± SD. *p<0.05 vs. control treatment or scrambled shRNA, determined using one-way ANOVA with Newman-Keuls post hoc correction for (A, B, D), and Student’s t-test for (F-N). Data in (A-N) were derived from three independent experiments.

LCN2 has been detected in pre-osteoblasts and osteoblasts^32^; however, whether osteocytes express LCN2 remains unclear. To determine whether osteocytes express Lcn2, we induced osteogenic differentiation in murine bone marrow stromal cells (BMSCs) using β-glycerophosphate and ascorbate. Pre-osteoblastic to mature osteoblastic marker *Runx2* and early osteocyte markers *Phex*, and *Dmp1* significantly increased by week two, whereas mature osteocyte markers *Fgf23* and *Sost* were detected only at weeks three and four (Fig. 1A). Notably, *Lcn2* mRNA levels progressively increased throughout differentiation, with mature osteocytes at weeks three and four exhibiting robust *Lcn2* expression (Fig. 1A). These findings were further validated in OCY454 osteocyte-like cells, a well-established in vitro model of osteocytes^37^. Differentiation of OCY454 cells for two weeks resulted in a marked increase in osteocyte markers *Dmp1, Phex*, and *Fgf23* expression (Fig. 1B), confirming their osteocytic identity. Consistently, *Lcn2* mRNA levels were upregulated during the differentiation of OCY454 cells (Fig. 1B), reinforcing that osteocytes are also a source of LCN2 in bone.

Since LCN2 binds iron-siderophore complexes in multiple tissues^26–31^, we investigated whether recombinant LCN2 increases intracellular Fe^2+^ levels under basal conditions. FerroOrange staining revealed a significant increase in Fe^2+^ levels in LCN2-treated cells, comparable to erastin, a known ferroptosis inducer (Fig. 1C, D). Given that intracellular iron accumulation is a key driver of ferroptosis, we next examined whether LCN2-induced iron overload contributes to oxidative stress and lipid peroxidation, two hallmarks of ferroptotic cell death. Flow cytometry analysis of DCFDA (2’,7’-dichlorodihydrofluorescein diacetate) fluorescence intensity demonstrated a significant increase in ROS production in LCN2-treated osteocytes compared to control treatment (Fig. 1E, F, Supplemental fig. S1A), indicating elevated oxidative stress. Similarly, BODIPY 581/591 C11 staining, which detects oxidized lipids via fluorescence shifts, showed that LCN2-treatment significantly increases oxidized BODIPY fluorescence, confirming heightened lipid peroxidation (Fig. 1G, H, Supplemental fig. S1B). To determine whether LCN2-driven iron overload, ROS accumulation, and lipid peroxidation culminate in ferroptotic cell death, we performed Annexin V/PI staining followed by flow cytometry. LCN2-treated osteocytes exhibited a significant increase in cell death, similar to erastin-treated cells (Fig. 1I, Supplemental fig. S1C, D), thus confirming LCN2 to be an inducer of ferroptosis in osteocytes.

To further validate LCN2’s role in ferroptosis, we transiently knocked down *Lcn2* in OCY454 cells using shRNA. Compared to scrambled control shRNA, *Lcn2* shRNA led to a 50% reduction in *LCN2* protein and mRNA levels (Fig. 1J, K). Notably, suppression of endogenous *Lcn2* alleviated oxidative stress, as evidenced by reduced DCFDA fluorescence intensity (Fig. 1L, Supplemental fig. S2A). Additionally, *Lcn2* knockdown decreased lipid peroxidation, shown by reduced BODIPY fluorescence (Fig. 1M, Supplemental fig. S2B), and lowered Annexin V/PI-positive cell populations, confirming that *Lcn2* knockdown protects osteocytes from ferroptotic cell death (Fig. 1N, Supplemental fig. S2C). Together, these findings establish that LCN2 promotes ferroptosis in osteocytes by driving iron accumulation, oxidative stress, and lipid peroxidation, ultimately leading to cell death. Furthermore, *Lcn2* knockdown mitigates these ferroptotic hallmarks, underscoring its critical role in osteocyte survival and iron homeostasis.

### LCN2 drives osteocyte ferroptosis via SLC22A17 under basal and iron overload conditions.

**Figure 2:**
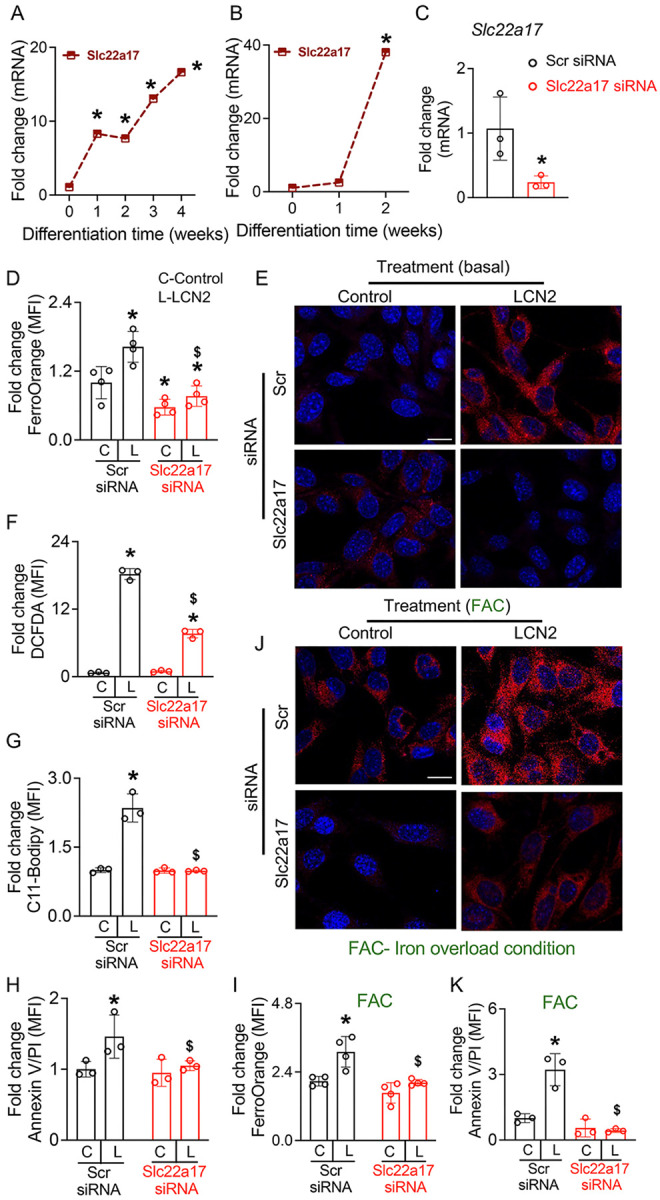
SLC22A17 mediates LCN2-induced iron accumulation, oxidative stress, lipid peroxidation, and ferroptosis in osteocytes. (A, B) qPCR analysis of Slc22a17 expression in (A) primary osteocytes derived from BMSCs and (B) differentiated OCY454 osteocyte-like cells. *p<0.05 vs. week 0, n=4 biological replicates/time point. (C) qPCR analysis of Slc22a17 knockdown efficiency in OCY454 cells transfected with scrambled control (Scr) siRNA or Slc22a17 siRNA for 72 hours. n=4 biological replicates/group. (D, E) Quantification (D) and representative image (E) of FerroOrange fluorescence (D) showing increased Fe^2+^ accumulation in OCY454 cells treated with rmLCN2 (100 ng/ml) for 48 hours, which is attenuated by Slc22a17 knockdown. (E) Representative FerroOrange staining images (scale bar = 40 μm). n=4 biological replicates/group. (F) Flow cytometric analysis of DCFDA fluorescence showing increased oxidative stress (ROS) in LCN2-treated OCY454 cells, which is reduced by Slc22a17 knockdown. (G) Flow cytometric quantification of C11-BODIPY fluorescence indicating increased lipid peroxidation in rmLCN2-treated OCY454 cells, which is attenuated by Slc22a17 knockdown. n=3 biological replicates/group. (H) Flow cytometric analysis of Annexin V/PI staining showing increased ferroptotic cell death in OCY454 cells treated with LCN2 for 48 hours, which is reduced by Slc22a17 knockdown. n=3 biological replicates/group. (I-K) Under iron overload conditions (FAC, 2.5 mM), Slc22a17 knockdown reduces Fe^2+^ accumulation (I, J) and ferroptotic cell death (K) in OCY454 cells treated with LCN2, n=3–4 biological replicates/group, scale bar in J = 40 μm. Data are presented as mean ± SD. *p<0.05 vs. control treatment; ^$^p<0.05 vs. LCN2-treated Scr siRNA, determined using one-way ANOVA with Newman-Keuls post hoc correction (A, B, D), Student’s t-test (C), or two-way ANOVA with Newman-Keuls post-hoc correction (F-K). Data in (A-K) were derived from three independent experiments. *Panels A and B contain data obtained from the experiment reported in* Figure 1A and 1B.

LCN2-bound iron-siderophores are internalized through receptor-mediated endocytosis^52^, prompting us to examine whether osteocytes express the LCN2 receptor, SLC22A17. We assessed *Slc22a17* levels by qPCR in matched RNA samples from murine BMSCs undergoing osteogenic differentiation and differentiated OCY454 osteocyte-like cells of Fig. 1A and 1B. During osteogenic differentiation of BMSCs, *Slc22a17* mRNA levels progressively increased, with a sharp upregulation at weeks three and four, coinciding with the transition of osteoblasts into mature osteocytes (Fig. 2A). Similarly, differentiated OCY454 cells exhibited a marked increase in Slc22a17 expression (Fig. 2B). To determine whether SLC22A17 is essential for LCN2-driven ferroptosis, we first silenced *Slc22a17* using siRNA (Fig. 2C) and then treated cells with recombinant LCN2. Cells pre-treated with scrambled siRNA had markedly increased intracellular Fe^2+^ levels in response to LCN2 and determined by FerroOrange staining. However, this effect was significant blunted by *Slc22a17* silencing (Fig. 2D, E). Similarly, recombinant LCN2-induced oxidative stress and lipid peroxidation were significantly upon *Slc22a17* knockdown (Fig. 2F, G). These findings demonstrate that LCN2-driven ferroptosis in osteocytes requires SLC22A17, and that SLC22A17 depletion mitigates iron accumulation, oxidative damage, and cell death.

While LCN2 promotes ferroptosis under basal conditions, we next investigated whether LCN2 amplifies osteocyte ferroptosis in an iron overload environment. Using ferric ammonium citrate (FAC), an established iron overload model^53,54^, we assessed LCN2’s impact on Fe^2+^ accumulation and cell death. FerroOrange staining confirmed that FAC alone elevated Fe^2+^ levels by 2-fold compared to basal conditions (Fig. 2D vs. 2I), and LCN2 further intensified Fe^2+^ accumulation beyond FAC alone (Fig. 2I, J, K). Similarly, FAC alone induced ferroptotic death in osteocytes, but LCN2 dramatically enhanced the susceptibility of osteocytes to FAC-induced ferroptosis, suggesting a compounding effect. Notably, *Slc22a17* knockdown mitigated these effects, reducing Fe^2+^ accumulation and ferroptotic death, further reinforcing that LCN2-driven iron uptake in osteocytes requires SLC22A17 under both basal and iron overload conditions. These findings establish that LCN2 exacerbates ferroptotic death under both basal and iron-overload conditions and that SLC22A17 as a key mediator of LCN2-driven Fe^2+^ uptake, oxidative stress, and ferroptosis in osteocytes.

### LCN2 Deletion from Osteocytes Reduces Ferroptosis Susceptibility by Limiting Iron Accumulation and Enhancing Antioxidant Defenses In Vivo.

**Figure 3:**
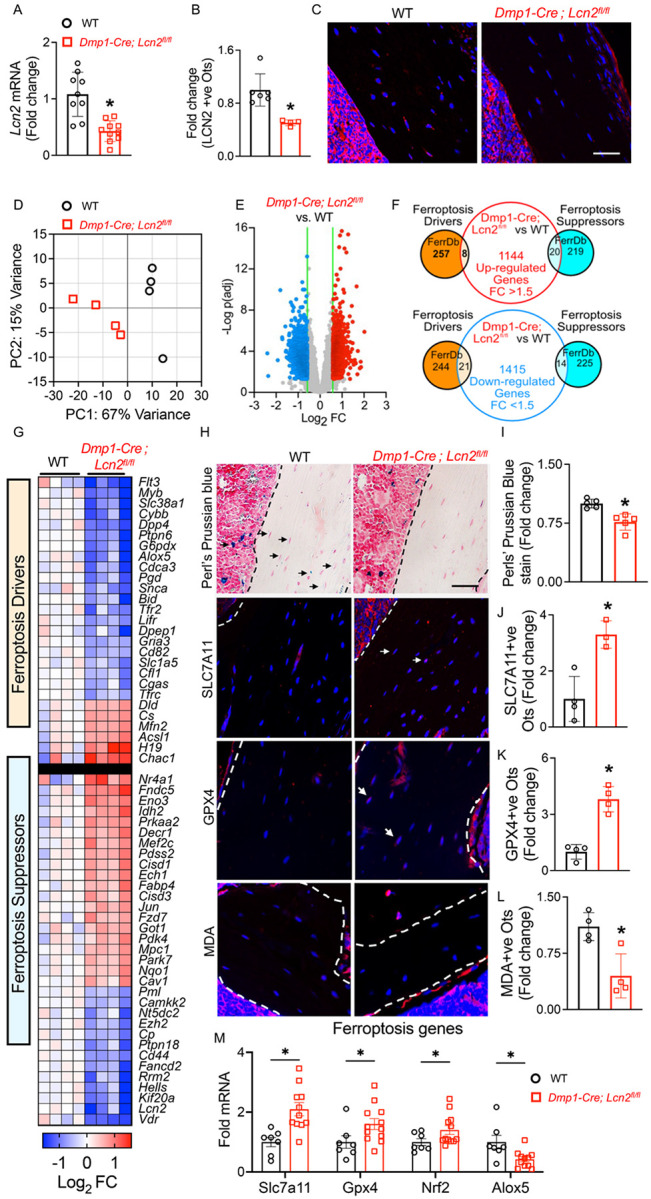
LCN2 deletion in osteocytes reduces iron accumulation and ferroptosis susceptibility in vivo. (A) qPCR analysis of Lcn2 expression in humeri from 13-week-old *Dmp1-Cre; Lcn2*^*fl/fl*^ and WT male mice confirms reduced Lcn2 mRNA levels in *Dmp1-Cre; Lcn2*^*fl/fl*^ bones. n=8–10 mice/group. (B, C) Quantification of LCN2 protein levels in osteocytes from *Dmp1-Cre; Lcn2*^*fl/fl*^ and WT tibial sections using immunofluorescence. n=3–6 mice/group, 4 ROI/mouse. (C) Representative immunofluorescence images of LCN2 (red) and DAPI (blue)-positive osteocytes in tibial sections from *Dmp1-Cre; Lcn2*^*fl/fl*^ and WT mice. Scale bar = 50 μm. (D, E) Principal component analysis (PCA) (D) and volcano plot (E) of RNA-seq data from humeri of *Dmp1-Cre; Lcn2*^*fl/fl*^ and WT mice, showing distinct clustering and differential gene expression (FDR < 0.05, FC ≥1.5). n=4 mice/group. (F, G) overlap between differentially expressed genes (1144 upregulated, 1415 downregulated) and ferroptosis-related genes from FerrDb, highlighting ferroptosis drivers and suppressors, was performed as shown in Venn diagram (F). Heatmap shows significant differentially expressed ferroptosis driver and suppressor genes from RNA-seq data in WT and *Dmp1-Cre; Lcn2*^*fl/fl*^ mouse bones (G). (H) Representative images of tibial sections from *Dmp1-Cre; Lcn2*^*fl/fl*^ and WT mice shows Perls’ Prussian blue stain and immunofluorescence for ferroptosis markers SLC7A11, GPX4, and MDA in osteocytes. (I) Quantification of Perls’ Prussian blue indicating Fe^3+^ deposits (blue) around osteocytes confirms reduced iron accumulation in *Dmp1-Cre; Lcn2*^*fl/fl*^ tibias. n=4 mice/group, 4 ROI/mouse. (J-L) Quantification of ferroptosis markers SLC7A11 (J), GPX4 (K), and MDA (L) in osteocytes from tibial sections of *Dmp1-Cre; Lcn2*^*fl/fl*^ and WT mice. White arrows indicate osteocytes positive for DAPI and the respective markers. n=4 mice/group, 4 ROI/mouse. (M) qPCR analysis of ferroptosis regulators showing upregulated ferroptosis suppressors (*Slc7a11, Gpx4, Nrf2*) and downregulated ferroptosis driver (Alox5) in *Dmp1-Cre; Lcn2*^*fl/fl*^ humeri. n=8–10 mice/group. Data are shown as mean ± SD, *p<0.05 compared to WT, determined using Student’s t-test (A, B, I-M). Scale bar for H= 50 μm.

To investigate the role of LCN2 in osteocytes in vivo, we generated *Dmp1-Cre; Lcn2*^*fl/fl*^ mice. qPCR analysis confirmed a significant reduction in *Lcn2* mRNA expression in bones of *Dmp1-Cre; Lcn2*^*fl/fl*^ mice compared to wild-type (WT) controls (Fig. 3A). Immunofluorescent staining further demonstrated a marked decrease in LCN2-positive osteocytes in *Dmp1-Cre; Lcn2*^*fl/fl*^ mice (Fig. 3B and 3C), validating efficient gene ablation.

To assess global transcriptional changes associated with osteocyte deficiency of *Lcn2*, we performed bulk RNA sequencing (RNA-seq) on bones from WT and *Dmp1-Cre; Lcn2*^*fl/fl*^ mice. RNA was extracted after removing epiphyses, bone marrow, and periosteum, to focus on osteocyte-enriched transcripts. Principal component analysis (PCA) revealed distinct clustering between individuals of WT and *Dmp1-Cre; Lcn2*^*fl/fl*^ groups (Fig. 3D), suggesting distinct transcriptional profiles. Differential expression analysis identified 2,593 differentially expressed genes (DEGs) with FDR < 0.05 and fold-change > 1.5 (Fig. 3E).

Since our in vitro findings suggested a crucial role for LCN2 in osteocyte ferroptosis, we next examined whether LCN2 ablation in osteocytes alters the expression of ferroptosis-related genes (FRGs) in the RNA-seq dataset. Cross-referencing our DEGs with FRGs curated from FerrDb, a publicly available database of validated ferroptosis regulators^42^, revealed that 21 ferroptosis driver genes were downregulated in *Dmp1-Cre; Lcn2*^*fl/fl*^ bones, while 20 ferroptosis suppressor genes were upregulated (Fig. 3F). A heatmap of differentially expressed ferroptosis-associated genes further illustrated this shift in gene expression (Fig. 3G). These transcriptional changes suggest that LCN2 deficiency may influence osteocyte resistance to ferroptosis.

To experimentally validate findings from RNA-seq, we assessed whether LCN2 ablation affects ferroptotic susceptibility in vivo. Given that ferroptosis is an iron-dependent form of cell death^55^, we first examined iron deposition in bone using Perl’s Prussian blue staining^43^, which detects ferric iron accumulation. Staining revealed a notable reduction in Fe^3+^ deposits in the cortical and trabecular bone osteocytes of *Dmp1-Cre; Lcn2*^*fl/fl*^ bones compared to WT controls (Fig. 3H, I), indicating that LCN2 deletion may help regulate intracellular iron loading in osteocytes, a key prerequisite for limiting ferroptotic vulnerability.

Since ferroptosis is linked to iron-induced lipid peroxidation^55^, we next examined key antioxidant regulators of ferroptosis suppression. Solute carrier family 7 member 11 (SLC7A11) is a cystine/glutamate transporter that has been shown to counteract oxidative stress by enhancing glutathione biosynthesis^55^. Immunohistochemical staining demonstrated a significant increase in SLC7A11 + ve osteocytes in *Dmp1-Cre; Lcn2*^*fl/fl*^ bones (Fig. 3H, J), suggesting enhanced cystine uptake and glutathione biosynthesis, which help counteract oxidative stress. Similarly, glutathione oxidase 4 (GPX4), a key ferroptosis inhibitor that neutralizes lipid peroxides^56^, was significantly upregulated in LCN2-deficient osteocytes (Fig. 3H, K). In accordance with reduced lipid peroxidation, we also observed reduced malondialdehyde (MDA), a well-established marker of lipid peroxidation and ferroptosis, in LCN2-deficient osteocytes (Fig. 1H, L). Collectively, these data reinforce the notion that LCN2 deletion protects osteocytes not only from iron-induced oxidative stress but also lipid peroxidation.

Supporting these observations, qPCR analysis revealed increased expression of *Slc7a11, Gpx4*, and *Nrf2*, a transcription factor and master regulator of the antioxidant response, in LCN2-deficient bones, indicating that LCN2 deletion enhances antioxidant defenses that suppress ferroptosis (Fig. 3M). Additionally, Alox5 (arachidonate 5-lipoxygenase), an enzyme that catalyzes lipid peroxidation and promotes ferroptosis^57^, was significantly downregulated in LCN2-deficient bones (Fig. 3M). Given that peroxidation of polyunsaturated fatty acids (PUFA) is essential for ferroptosis initiation, reduced Alox5 expression further supports that LCN2 deletion limits ferroptotic susceptibility by restricting lipid peroxidation. While *Alox5* was detected as a differentially expressed gene in RNA-seq, transcriptional changes in *Slc7a11, Gpx4*, and *Nrf2* were not detected, which could be a caveat of small sample size or differences in sensitivity between RNA-seq and qPCR methodologies. Regardless, these findings provide experimental evidence that LCN2 ablation in osteocytes is associated with reduced ferroptotic susceptibility. By potentially lowering iron accumulation, supporting ferroptosis suppressor pathways, and downregulating lipid peroxidation drivers, LCN2 deletion may contribute to a protective phenotype against ferroptotic stress in osteocytes.

### LCN2 Ablation Enhances Mitochondrial Function in Osteocytes.

Mitochondria play a critical role in cellular energy metabolism, redox balance, and iron homeostasis, making them particularly vulnerable to iron overload and oxidative stress^58–61^. Given that LCN2 functions as an iron-trafficking protein, and excessive iron is known to disrupt mitochondrial integrity—leading to membrane depolarization, dysregulated oxidative phosphorylation (OXPHOS), and ATP depletion—we investigated whether LCN2 deletion in osteocytes improves mitochondrial function in osteocytes under basal conditions.

**Figure 4:**
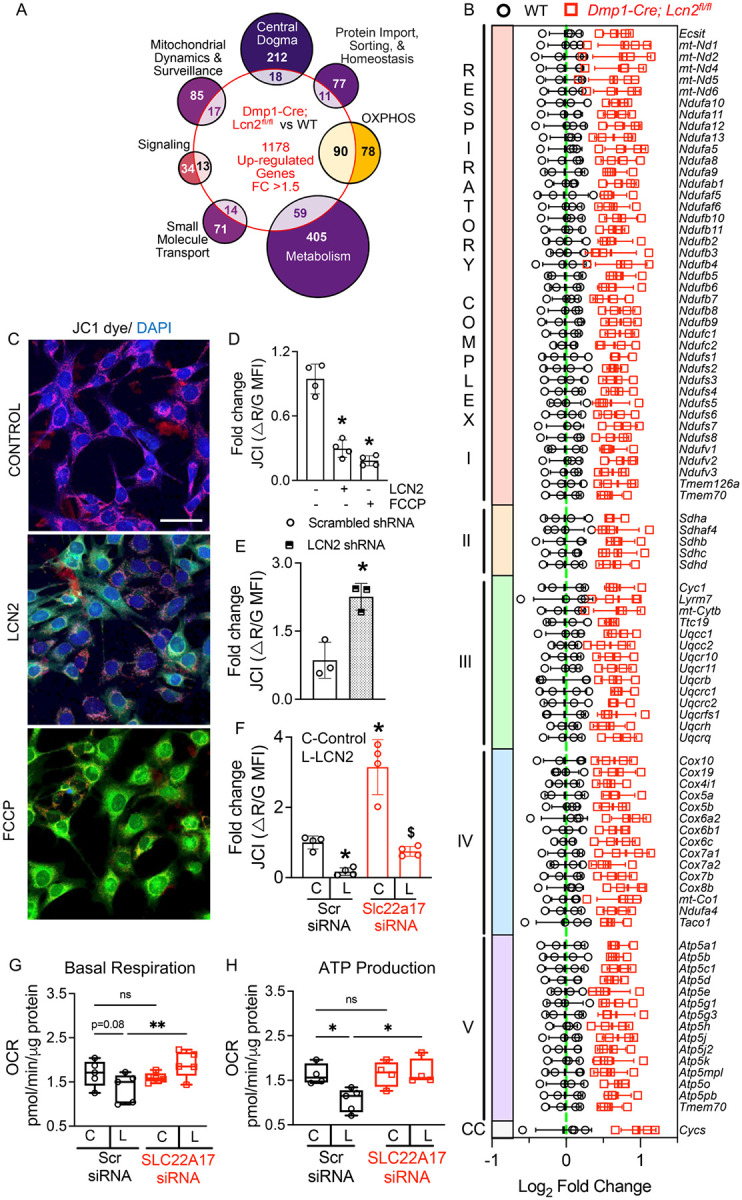
LCN2 deletion enhances mitochondrial function in osteocytes by preserving membrane potential and oxidative phosphorylation. (A) Bulk RNA-seq analysis of humeri from *Dmp1-Cre; Lcn2*^*fl/fl*^ and WT mice shows upregulation of mitochondrial-related pathways, cross-referenced with MitoCarta3.0. Venn diagram highlights differentially expressed genes across mitochondrial pathways, including central dogma, protein import, oxidative phosphorylation (OXPHOS), metabolism, small molecule transport, signaling, and mitochondrial dynamics. n=4 mice/group. (B) Heatmap displaying differentially expressed genes in respiratory complexes I-V of the electron transport chain (ETC), showing broad upregulation of mitochondrial respiration-related genes in *Dmp1-Cre; Lcn2*^*fl/fl*^ bones compared to WT mice. (C) Representative JC1 staining images of OCY454 osteocyte-like cells treated with control (aMEM), rmLCN2 (100 ng/ml), or FCCP (positive control) for 24 hours. Scale bar = 40 μm. (D) Quantification of JC1 fluorescence shows decreased mitochondrial membrane potential (ΔΨm) in rmLCN2-treated osteocytes, similar to the mitochondrial uncoupler FCCP. n=4 biological replicates. (E) LCN2 shRNA rescues mitochondrial membrane potential, as indicated by JC1 fluorescence quantification in OCY454 cells transfected with scrambled shRNA or *Lcn2* shRNA. n=3 biological replicates. (F) Knockdown of Slc22a17 prevents LCN2-induced mitochondrial depolarization in OCY454 cells transfected with scrambled control (Scr) siRNA or Slc22a17 siRNA and treated with rmLCN2. n=4 biological replicates. (G, H) Seahorse extracellular flux analysis shows that LCN2 treatment does not significantly alter basal respiration (G), but impairs ATP production (H), which is restored upon Slc22a17 knockdown. n=6 biological replicates/group. Data are shown as mean ± SD. *p<0.05 vs. control treatment; ^$^p<0.05 vs. LCN2-treated Scr siRNA, determined using either one-way ANOVA with Newman-Keuls post hoc correction (D), Student’s t-test (E), or two-way ANOVA with Newman-Keuls post hoc correction (F-H). Data in D-H were reproduced in three independent experiments.

To assess transcriptional changes, we cross-referenced 1,178 upregulated DEGs from *Dmp1-Cre; Lcn2*^*fl/fl*^ vs. WT mouse bone RNA-seq with MitoCarta 3.0, a curated database of mitochondrial-localized genes^62^. This analysis revealed an enrichment of pathways associated with mitochondrial dynamics, transcription and translation, protein import/homeostasis, OXPHOS, and metabolism (Fig. 4A). Strikingly, nearly half of all genes related to OXPHOS were upregulated in bones with LCN2-deficient osteocytes, including genes of electron transport chain (ETC) complexes I-V (Fig. 4B). Additionally, many genes associated with mitochondrial integrity, to include stability, fusion, and mitophagy, were increased in bones of *Dmp1-Cre; Lcn2*^*fl/fl*^ mice (Supplemental fig S3A). To corroborate whether these transcriptomic changes observed *in vivo* correspond to functional improvements in mitochondria in osteocytes, we assessed mitochondrial membrane potential (ΔΨm) using JC1 dye staining, a widely used indicator of mitochondrial integrity. LCN2-treated OCY454 cells exhibited increased mitochondrial depolarization, as indicated by a shift from JC1 aggregates (red) to JC1 monomers (green) (Fig. 4C). Quantification confirmed a significant reduction in JC1 fluorescence, similar to the effect of carbonyl cyanide-p-trifluoromethoxy phenylhydrazone (FCCP), a mitochondrial uncoupler (Fig. 4D). Consequently, we found that *LCN2* adversely affected mitochondrial OXPHOS function (Supplemental fig S3B). Indeed, *Lcn2* knockdown via shRNA restored mitochondrial membrane potential, as evidenced by increased JC1 fluorescence (Fig. 4E). These findings suggest that endogenous LCN2 contributes to mitochondrial dysfunction in osteocytes even under basal conditions.

Next, we examined whether recombinant LCN2-induced mitochondrial dysfunction occurs via SLC22A17. JC1 fluorescence showed that knockdown of *Slc22a17* significantly attenuated LCN2-induced mitochondrial depolarization (Fig. 4F). Lastly, we found that LCN2-SLC22A17 signaling affects mitochondrial respiration using Seahorse extracellular flux analysis. Both basal respiration (p < 0.08, Fig. 4G) and ATP production (Fig. 4H) decreased following LCN2 treatment. However, *Slc22a17* knockdown decreased the effect of LCN2 on basal respiration and ATP production (Fig. 4G, H). Collectively, our data identify LCN2-SLC22A17 signaling as a novel regulator of mitochondrial dysfunction and suggest that targeting this pathway may enhance mitochondrial activity at baseline and reduce mitochondrial stress and ferroptotic susceptibility under adverse conditions.

### LCN2 Suppresses Wnt/β-Catenin Signaling in Osteocytes.

**Figure 5:**
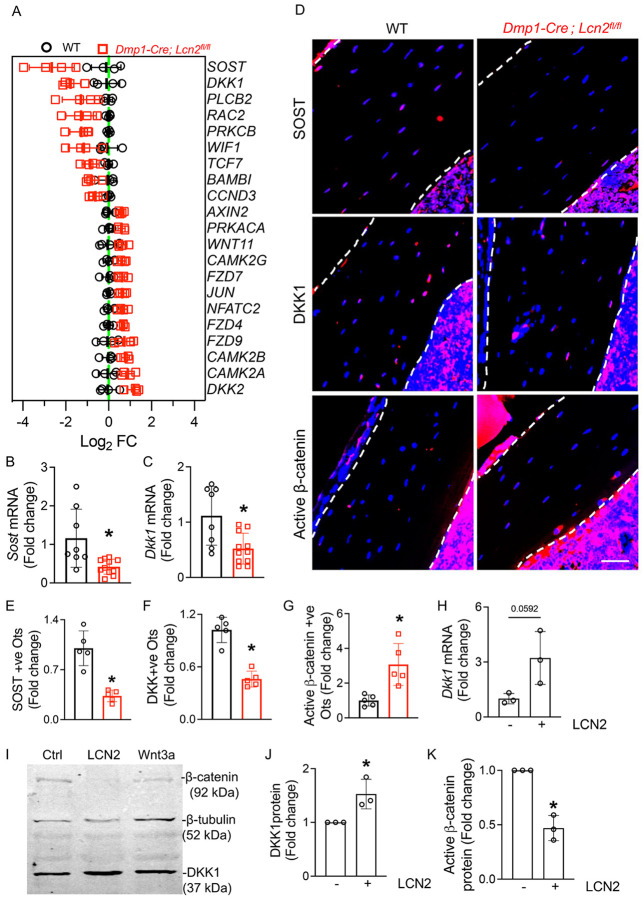
LCN2 regulates canonical Wnt/β-catenin signaling by modulating SOST and DKK1 expression in osteocytes. (A) Heatmap of RNA-seq data from humeri of *Dmp1-Cre; Lcn2*^*fl/fl*^ and WT mice showing differential expression of Wnt signaling-related genes. n=4 mice/group. (B, C) qPCR analysis of Sost (B) and Dkk1 (C) mRNA expression in humeri of *Dmp1-Cre; Lcn2*^*fl/fl*^ and WT mice. n=8–10 mice/group. (D) Representative immunofluorescence images of tibial osteocytes showing reduced SOST and DKK1 expression and increased active β-catenin staining in *Dmp1-Cre; Lcn2*^*fl/fl*^ mice. Scale bar = 50 μm. (E-G) Quantification of immunofluorescence staining shows decreased SOST-positive (E, SOST+ve) and DKK1-positive (F, DKK1+ve) osteocytes, while active β-catenin-positive (G) osteocytes are increased in *Dmp1-Cre; Lcn2*^*fl/fl*^ bones. n=5 mice/group, 4 ROI/mouse. (H) qPCR analysis of Dkk1 mRNA expression in OCY454 osteocyte-like cells treated with control (aMEM) or rmLCN2 (100 ng/ml) for 24 hours. n=3 biological replicates/group. (I) Representative western blot of β-catenin and DKK1 protein expression in OCY454 cells treated with rmLCN2 or Wnt3a. (J, K) Quantification of western blot data shows increased DKK1 (J) and reduced active β-catenin (K) protein levels following rmLCN2 treatment. n=3 biological replicates. Data are presented as mean ± SD. *p<0.05 vs. WT mice, determined using Student’s t-test (B, C, E-H, J-K). Data in (H-K) were reproduced in at least two independent experiments.

Recent studies suggest a strong link between mitochondrial biogenesis and Wnt/β-catenin signaling. Activation of mitochondrial OXPHOS in bone marrow stromal cells has been shown to enhance osteogenic differentiation by promoting β-catenin acetylation and activity^63^. Additionally, overexpression of gene encoding for transcription factor A, mitochondrial (Tfam) protein, a key regulator of mitochondrial transcription, was found to enhance Wnt-induced osteogenesis^64^. Given our observation that LCN2 deficiency in osteocytes improved mitochondrial integrity and function *in vitro*, we hypothesized that *Lcn2* deletion in osteocytes may enhance Wnt/β-catenin signaling, a critical pathway for bone formation. To test this, we analyzed transcriptomic data from *Dmp1-Cre; Lcn2*^*fl/fl*^ and WT bones for differentially expressed genes related to Wnt/β-catenin signaling. Notably, negative regulators of Wnt signaling, Sclerostin (*Sost)* and Dickkopf-1 (*Dkk1*), were significantly downregulated in LCN2-deficient bones (Fig. 5A). We validated reduced *Sost* and *Dkk1* expression in independent bone samples of *Dmp1-Cre; Lcn2*^*fl/fl*^ mice relative to WT mice (Fig. 5B, C). To assess whether these transcriptomic changes translated to protein-level alterations, we performed immunofluorescence staining for SOST and DKK1 in bone sections. Consistent with RNA-seq and qPCR results, bones of *Dmp1-Cre; Lcn2*^*fl/fl*^ mice displayed significantly fewer SOST-positive (Fig. 5D, E) and DKK1-positive osteocytes (Fig. 5D, F). Since SOST and DKK1 inhibit β-catenin activity, we next investigated whether LCN2 deletion enhances Wnt signaling. Indeed, LCN2-deficient bones exhibited a significant increase in active β-catenin-positive osteocytes (Fig. 5D, G), suggesting that LCN2 deficiency is associated with elevated Wnt/β-catenin signaling in osteocytes.

To directly examine whether LCN2 inhibits Wnt signaling in a cell-autonomous manner, we treated OCY454 osteocyte-like cells with recombinant LCN2. qPCR revealed a trend toward increased *Dkk1* mRNA following LCN2 treatment (Fig. 5H, p = 0.0592), while western blot confirmed LCN2 upregulates DKK1 at the protein level (Fig. 5I, J) and reduces active β-catenin expression (Fig. 5I, K). Together, these findings demonstrate that LCN2 suppresses Wnt/β-catenin signaling by upregulating DKK1 and SOST. This LCN2-mediated suppression of Wnt may impair osteocyte function and contribute to bone loss in skeletal diseases.

### LCN2 Deletion Specifically Enhances Trabecular Bone Mass By Increasing Bone Formation.

**Figure 6:**
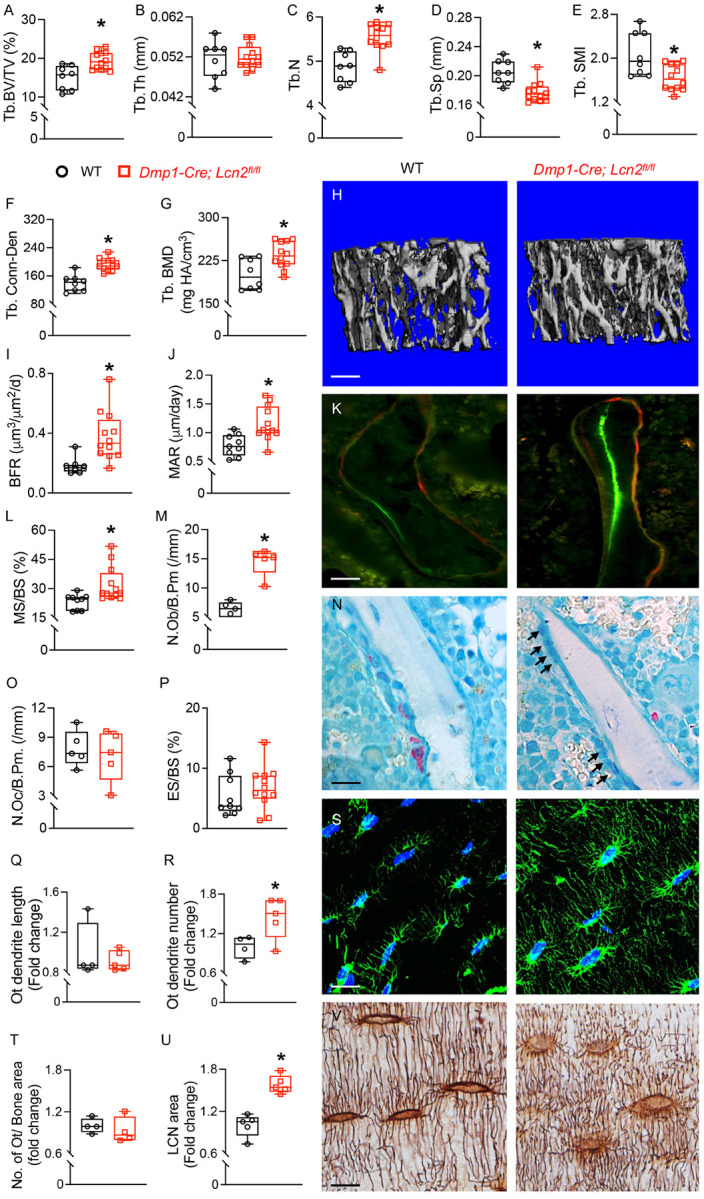
LCN2 deletion enhances trabecular bone mass, osteoblast activity, and osteocyte dendritic connectivity. (A-G) μCT analysis of femurs from 13-week-old male *Dmp1-Cre; Lcn2*^*fl/fl*^ and WT mice. Trabecular bone volume fraction (BV/TV, A), trabecular thickness (Tb.Th, B), trabecular number (Tb.N, C), trabecular spacing (Tb.Sp, D), structure model index (Tb.SMI, E), connectivity density (Tb.Conn-Den, F), and trabecular bone mineral density (Tb.BMD, G) were measured. Representative 3D reconstruction of trabecular bone shown (H). n=8–10 mice/group, scale bar = 100 μm. (I-L) Dynamic histomorphometry of tibias from *Dmp1-Cre; Lcn2*^*fl/fl*^ and WT mice shows bone formation rate (BFR, I), mineral apposition rate (MAR, J), and mineralizing surface per bone surface (MS/BS, L). Representative calcein and alizarin red double labeling images is shown (K). n=8–10 mice/group, scale bar = 50 μm. (M-P) Static histomorphometry quantifies osteoblast number per bone perimeter (N.Ob/B.Pm, M), osteoclast number per bone perimeter (N.Oc/B.Pm, O), and eroded surface per bone surface (ES/BS, P) in toluidine blue and TRAP-stained tibias. Representative images of osteoblasts (N, black arrows) and osteoclasts (red cell on bone surface). n=6 mice/group, 16 ROIs/mouse, scale bar = 50 μm. (Q-S) Phalloidin staining of osteocytes shows increased dendrite length (Q) and dendrite number (R) in *Dmp1-Cre; Lcn2*^*fl/fl*^ tibias. Representative phalloidin (green) and DAPI (blue) staining shown in (S). n=4–5 mice/group, 4 ROIs/mouse, scale bar = 20 μm. H&E staining quantifies osteocyte number per bone area (T), while Ploton silver nitrate staining visualizes the lacunocanalicular network (U, V), n=4–5 mice/group, 4 ROIs/mouse, scale bar = 20 μm. Data are presented as mean ± SD. *p<0.05 vs. WT mice, determined using Student’s t-test.

To evaluate the impact of *Lcn2* deletion in osteocytes on bone architecture, we conducted micro-computed tomography (μCT) analysis on *Dmp1-Cre; Lcn2*^*fl/fl*^ and WT mice. Loss of *Lcn2* in osteocytes significantly increased bone volume fraction (BV/TV) and trabecular number (Tb. N) (Fig. 6A–C), along with reduced trabecular spacing (Tb. Sp) (Fig. 6D). Additionally, the structure model index (Tb. SMI) was lower (Fig. 6E,H), suggesting a shift toward a more plate-like, mechanically advantageous trabecular structure (Table 1).

Since, osteocytes are critical regulators of osteoblasts and osteoclasts, we assessed whether increased trabecular bone mass in *Dmp1-Cre; Lcn2*^*fl/fl*^ mice was a result of enhanced osteoblast or reduced osteoclast activity in bone using dynamic and static histomorphometry. Calcein and Alizarin Red double labeling revealed increased bone formation rate (BFR), mineral apposition rate (MAR), and mineralized surface per bone surface (MS/BS) (Fig. 6I–L) in bones of *Dmp1-Cre; Lcn2*^*fl/fl*^ mice. Consistently, static histomorphometry showed an increased number of osteoblasts per bone perimeter (N.Ob/B.Pm) (Fig. 6M), while osteoclast number and activity remained unchanged, suggesting that LCN2 deletion enhances trabecular bone mass by promoting osteoblast function. We further examined osteocyte number, connectivity, and lacunocanalicular network integrity. Phalloidin and Ploton silver nitrate staining revealed no change in osteocyte dendrite length (Fig. 6Q) but increased dendrite number (Fig. 6R, S) in bones of *Dmp1-Cre; Lcn2*^*fl/fl*^ mice, along with a larger lacunocanalicular network (LCN area, Fig. 6T, U, V). However, osteocyte number in trabecular and cortical bone remained unchanged between genotypes.

**Figure 7:**
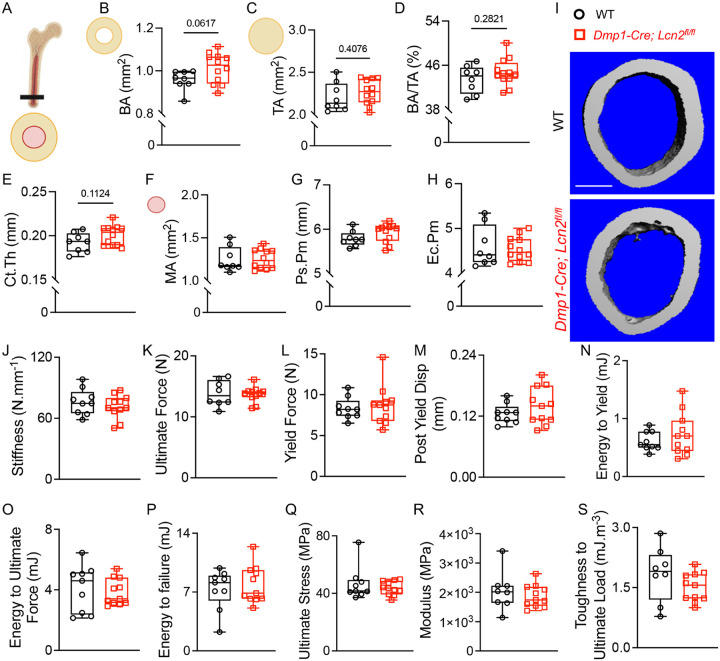
LCN2 deletion does not alter cortical bone structure or mechanical properties. (A) Schematic representation of the cortical bone cross-section showing analyzed parameters. (B-H) μCT analysis of femurs from 13-week-old male *Dmp1-Cre; Lcn2*^*fl/fl*^ and WT mice. Cortical bone area (BA, B), total area (TA, C), cortical bone mass (BA/TA, D), cortical thickness (Ct.Th, E), marrow area (MA, F), periosteal perimeter (Ps.Pm, G), and endocortical perimeter (Ec.Pm, H) were analyzed. Representative μCT cross-sectional images of cortical bone are shown in (I). n=8–10 mice/group, scale bar = 100 μm. (J-S) Three-point bending mechanical testing of femurs from 17-week-old *Dmp1-Cre; Lcn2*^*fl/fl*^ and WT male mice. Structural and material properties including stiffness (J), ultimate force (K), yield force (L), post-yield displacement (M), energy to yield (N), energy to ultimate force (O), energy to failure (P), ultimate stress(Q), modulus (R), and toughness to ultimate load (S) remained comparable between genotypes. Data are presented as mean ± SD. n=8–10 mice/group, *p<0.05 vs. WT mice, determined using Student’s t-test.

Cortical bone analysis revealed a trend toward increased cortical bone area (BA, Fig. 7A, B) and total area (TA, Fig. 7C) in *Dmp1-Cre; Lcn2*^*fl/fl*^ mice, though differences did not reach statistical significance (p = 0.0617 and p = 0.4076, respectively). As a result, cortical bone mass (BA/TA, Fig. 7D, E) remained unchanged (p = 0.2821). Similarly, cortical thickness (Ct. Th, Fig. 7F) showed a modest but non-significant increase (p = 0.1124), while marrow area (MA, Fig. 7G), periosteal perimeter (Ps. Pm, Fig. 7H), and endocortical perimeter (Ec. Pm, Fig. 7I) were comparable between genotypes. Overall, LCN2 deletion had no significant effect on cortical bone morphology (Table 1). The differential impact of osteocytic-LCN2 ablation on trabecular and cortical bone morphometry was also observed in tibias of *Dmp1-Cre; Lcn2*^*fl/fl*^ mice (Supplemental fig. S4). To determine whether deletion of *Lcn2* in osteocytes affects cortical bone mechanical properties, we conducted three-point bending tests. Structural properties of stiffness (Fig. 7J), ultimate force (Fig. 7K), yield force (Fig. 7L), post-yield displacement (Fig. 7M), and energy to yield (Fig. 7N) were comparable across genotypes. Similarly, material properties—including energy to failure (Fig. 7P), ultimate stress (Fig. 7Q), elastic modulus (Fig. 7R), and toughness to ultimate load (Fig. 7S)—remained unchanged (Table 2). The lack of a cortical bone phenotype in *Dmp1-Cre; Lcn2*^*fl/fl*^ mice suggests that osteocyte intrinsic LCN2 regulates bone remodeling indirectly through osteoblasts, which are more abundant in trabecular bone. Since cortical bone undergoes slower remodeling and is more responsive to mechanical loading, future studies incorporating mechanical load could reveal a more pronounced cortical bone response to LCN2 ablation.

## Discussion

Iron homeostasis is essential for skeletal integrity^6^, yet the mechanisms governing non-transferrin-bound iron (NTBI) transport in bone cells under physiological or iron-overload conditions remain poorly understood. While transferrin-bound iron uptake via transferrin receptor 1 (TFR1) is well characterized, alternative iron import pathways in bone cells—particularly in osteocytes, the most abundant and functionally dominant regulators of bone remodeling— have not being explored. Our study identifies LCN2-SLC22A17 as a novel NTBI uptake mechanism in osteocytes. We demonstrate that LCN2 binds SLC22A17 to facilitate intracellular Fe^2+^ accumulation, thereby modulating osteocyte susceptibility to ferroptosis, an iron-dependent form of regulated cell death. Additionally, we show that LCN2 is a negative regulator of mitochondrial integrity and function in osteocytes and that, conversely, LCN2 ablation in osteocytes enhances osteocyte-driven osteoblastic activity, leading to increased trabecular bone mass. Our findings reveal a novel autocrine function of osteocyte-derived LCN2 with direct consequences for osteocyte function and skeletal homeostasis.

A key finding of our study is that LCN2-SLC22A17 signaling facilitates iron uptake under both basal and iron-overload conditions. While LCN2 exists in holo (iron-bound) and apo (iron-free) forms, its role in iron mobilization appears context-dependent^65^. Our data indicate that LCN2 primarily functions to increase intracellular iron levels. However, the precise mechanisms underlying LCN2-mediated Fe^2+^ accumulation remain incompletely understood. While our results demonstrate that SLC22A17 is required for LCN2-driven iron uptake, we cannot exclude the involvement of additional pathways. One possibility could be that LCN2 interacts with NCOA4 to promote ferritinophagy, where ferritin degradation liberates stored iron into the labile iron pool, amplifying ferroptotic susceptibility^66^. Notably, under iron overload conditions, we report that LCN2 further exacerbates osteocyte ferroptotic death, an effect mitigated by SLC22A17 depletion. Ablation of SLC22A17 reduces iron accumulation in osteocytes and confers resistance to ferroptosis, suggesting that targeting this receptor could provide a novel therapeutic approach for mitigating iron overload-induced skeletal damage. Given that SLC22A17 has broader metabolic roles, particularly in regulating tight junctions and systemic iron distribution^67,68^, future studies should examine its function beyond iron uptake in bone.

Beyond ferroptosis, our findings reveal that LCN2 also negatively impacts mitochondrial function in osteocytes. Mitochondria are essential for ATP production, redox balance, and iron homeostasis, and excess iron has been shown to disrupt mitochondrial respiration, leading to depolarization, ATP depletion, and increased ROS levels. We found that LCN2 compromises mitochondrial integrity, exacerbating oxidative stress and reducing osteocyte viability. Conversely, osteocytes lacking LCN2 exhibit increased expression of mitochondrial biogenesis genes, suggesting improved mitochondrial function in the absence of LCN2. RNA-seq analysis further reveals upregulation of Mtfp1, a key regulator of mitochondrial fusion and inner membrane stability, as well as genes involved in cardiolipin biosynthesis^69^.

Interestingly, LCN2 ablation was associated with elevated cardiolipin species enriched in long-chain polyunsaturated fatty acids (LC-PUFAs) and reduced levels of those containing monounsaturated fatty acids^70^. These shifts in cardiolipin composition may influence mitochondrial membrane properties and activate compensatory pathways such as peroxisomal metabolism and mTOR signaling, thereby promoting cellular resilience to metabolic stress^30,70^. Although the metabolic programming of osteocytes remains poorly defined, emerging evidence suggests that mitochondrial integrity plays a critical role in mechanotransduction and angiogenesis^71,72^. Given that LCN2 expression is responsive to mechanical stimuli^34^, it is possible that the LCN2–SLC22A17 axis may influence osteocyte mitochondrial function and skeletal homeostasis under load, particularly in aging contexts, which warrants further investigation.

In addition to its role in osteocyte survival and connectivity, LCN2 appears to suppress bone formation by inhibiting Wnt/β-catenin signaling. The Wnt pathway is a crucial regulator of osteoblast differentiation and bone mass, and its disruption is a hallmark of osteoporosis^73^. Elevated expression of Wnt inhibitors, particularly Dickkopf-1 (DKK1), has been implicated in postmenopausal and age-related bone loss^74,75^. While therapies targeting sclerostin (SOST) are being explored for their anabolic effects on bone^76^. Our findings demonstrate that LCN2 upregulates both SOST and DKK1, thereby suppressing Wnt signaling and reducing osteoblast activity. Previous studies have reported a correlation between increased LCN2 and DKK1 serum levels in osteoporotic patients^75^; our study provides experimental validation of this relationship. However, the precise mechanism by which LCN2 regulates DKK1 remains to be determined. A recent report suggests that LCN2 may interact with LRP6, sequestering it away from Wnt ligands. Further studies employing co-immunoprecipitation and LRP6 localization assays in LCN2-treated osteocytes will be necessary to clarify whether LCN2 directly modulates Wnt signaling through LRP6.

LCN2’s effects on bone mass appear to be compartment-specific, as its deletion resulted in increased trabecular bone volume without significantly impacting cortical bone morphology. This is likely attributable to LCN2’s influence on osteoblast activity, which is more prominent in trabecular bone due to its higher remodeling rate. Importantly, LCN2 ablation led to increased osteocyte connectivity in both trabecular and cortical bone. While the osteocyte network has been recognized as a critical component of mechanosensation and bone anabolism, recent findings suggest that even in the absence of a fully intact network, osteocytes can still perceive and respond to mechanical load^77^. Surprisingly, despite enhanced osteocyte connectivity in LCN2-deficient mice, cortical bone morphology remained largely unchanged under basal conditions. Given that osteocytic LCN2 primarily regulates bone mass through osteoblasts, it is possible that its influence on cortical bone remodeling would be more apparent under mechanical loading conditions. Future studies incorporating mechanical loading models could reveal whether osteocytic LCN2 plays a more substantial role in cortical bone adaptation when subjected to increased biomechanical demands.

Moreover, considering LCN2’s diverse roles^27,52,78^, particularly in inflammatory signaling, defining the specific contribution of its receptor, SLC22A17, in mediating iron homeostasis and mitochondrial dysfunction could provide a targeted therapeutic strategy for mitigating ferroptosis-related bone loss. A deeper understanding of LCN2-SLC22A17 interactions using SLC22A17-floxed mice could clarify receptor-specific functions and identify new avenues for intervention. Additionally, given emerging evidence that LCN2’s iron-binding capacity may be leveraged for iron sequestration^79^, targeting SLC22A17 in iron-overload conditions could present a novel strategy for therapeutic iron depletion.

One limitation of our study is the lack of investigation into potential sex-specific differences in LCN2 function. LCN2 exhibits sexually dimorphic effects in multiple tissues, particularly in metabolic and reproductive contexts^80,81^, raising the possibility that its impact on osteocytes and bone remodeling may also differ between males and females. Since iron metabolism and skeletal regulation are known to be influenced by sex hormones, future studies should explore whether LCN2’s role in osteocyte function varies by sex, which could have important implications for developing targeted therapeutic approaches.

In summary, our study identifies LCN2 as a critical regulator of osteocyte ferroptosis, mitochondrial integrity, and Wnt signaling, highlighting its central role in osteocyte-mediated bone remodeling. A novel aspect of our findings is the demonstration that LCN2 exerts its effects on bone not solely through endocrine mechanisms, but also via autocrine and paracrine signaling pathways mediated by its receptor SLC22A17. These insights broaden the physiological relevance of LCN2 beyond its well-established roles in metabolism and inflammation, positioning it as a key modulator of skeletal homeostasis. Given the reported increase in circulating LCN2 levels with aging^75,82^, future studies are warranted to explore its potential contribution to age-related iron dysregulation and osteoporosis.

## Figures and Tables

**Table 1:** μCT analysis of femurs harvested from 13-week-old WT and *Dmp1-Cre; Lcn2*^*fl/fl*^ male mice shows significant changes in trabecular but not cortical bone parameters.

Parameters	WT	Dmp1-Cre; Lcn2^fl/fl^	p-value
**Trabecular Bone**			
TV, mm^3^	2.7 ± 0.271	2.818 ± 0.209	0.283
BV,mm^3^	0.407 ± 0.101	0.548 ± 0.082*	0.003
BS, mm^2^	21.004 ± 3.881	26.889 ± 3.034*	0.001
BV/TV, %	15.011 ±3.114	19.413 ± 2.315*	0.002
BS/TV, mm^−1^	7.753 ± 0.928	9.535 ± 0.693*	0.000
BS/BV, mm^−1^	52.707 ± 6.553	49.414 ± 3.289	0.152
Tb.N, mm^−1^	4.871 ± 0.343	5.548 ± 0.308*	0.000
Tb.Th, mm	0.051 ± 0.005	0.052 ± 0.003	0.557
Tb.Sp, mm	0.205 ± 0.016	0.178 ± 0.014*	0.001
SMI	2.071 ± 0.398	1.648 ± 0.235*	0.008
Conn.D, mm^−3^	139.831 ± 24.002	192.429 ± 17.597*	0.000
BMD, mgHA/ccm	201.115 ± 26.85	234.598 ± 22.891*	0.008
TMD, mgHA/ccm	1045.875 ± 14.897	1064.225 ± 18.698*	0.032
**Cortical Bone**			
Ct.Th, mm	0.192 ± 0.011	0.201 ± 0.011	0.112
Ct. Ar, mm^2^	0.957 ± 0.047	1.013 ± 0.07	0.062
Ma.Ar, mm^2^	1.25 ± 0.145	1.253 ± 0.112	0.950
Tt.Ar, mm^2^	2.206 ± 0.171	2.266 ± 0.144	0.408
Ct.Ar/Tt.Ar, %	43.496 ± 2.493	44.75 ± 2.47	0.282
Ps.Pm, mm	5.789 ± 0.172	5.922 ± 0.211	0.155
Ec.Pm, mm	4.594 ± 0.456	4.535 ± 0.273	0.720
BMD, mgHA/ccm	1219.126 ± 23.117	1238.694 ± 22.449	0.075
TMD, mgHA/ccm	1290.49 ± 24.592	1310.578 ± 19.281	0.056

**Table 2:** Flexural strength analysis of femurs harvested from 17-week-old WT and *Dmp1-Cre; Lcn2*^*fl/fl*^ male mice show no apparent changes in structural and material properties of bone.

Parameter	WT	Dmp1-Cre; Lcn2^fl/fl^	p-value
**Structural Properties**			
Energy to failure, mJ	7.663 ± 2.894	7.585 ± 1.691	0.941
Stiffness, N/mm	75.838 ± 12.304	71.621 ± 12.159	0.453
Ultimate Force, N	14.096 ± 2.11	13.6 ± 1.166	0.513
failure (breaking) force, N	11.722 ± 2.483	10.907 ± 1.759	0.402
Yield Force, N	9.033 ± 2.473	8.114 ± 1.383	0.307
Postyield Displacement, mm	0.585 ± 0.236	0.606 ± 0.186	0.825
Preyield displacement, mm	0.133 ± 0.026	0.133 ± 0.034	0.954
Postyield energy to failure, mJ	6.945 ± 2.735	6.948 ± 1.756	0.998
Energy to Yield, mJ	0.718 ± 0.325	0.637 ± 0.27	0.551
Energy to Ultimate Force, mJ	4.292 ± 1.584	3.686 ± 0.701	0.268
**Material Properties**			
Ultimate Stress, MPa	43.213 ± 4.26	46.56 ± 10.706	0.391
Modulus, MPa	1826.336 ± 342.224	2023.56 ± 596.731	0.392
Toughness to ultimate load, mJ/m^3^	1.711 ± 0.665	1.452 ± 0.319	0.481
Toughness by energy to failure, mJ/m^3^	3.098 ± 1.234	2.887 ± 0.55	0.896
Toughness by energy to yield, mJ/m^3^	0.284 ± 0.124	0.255 ± 0.11	0.657
Post-Yield Toughness, mJ/m^3^	2.814 ± 1.169	2.632 ± 0.585	0.857

## Data Availability

All data associated with this study are present in the paper or the supplemental information. Reagents associated with this study are available from the corresponding author. The RNA-seq data generated in this study have been deposited in the SRA database under accession code BioProject PRJNA1249006.
